# Case report: A creatine kinase-borg scale values-based approach to tailor physical training in a central core myopathy patient

**DOI:** 10.3389/fphys.2024.1404657

**Published:** 2024-07-23

**Authors:** Oscar Crisafulli, Jessica Lacetera, Giorgio Bottoni, Angela Berardinelli, Luca Grattarola, Martina Veltroni, Stefano Acquadro, Massimo Negro, Emanuela Lavaselli, Giuseppe D’Antona

**Affiliations:** ^1^ Criams-Sport Medicine Centre Voghera, University of Pavia, Voghera, Italy; ^2^ Child Neuropsychiatry, IRCCS Mondino Foundation, Pavia, Italy; ^3^ Department of Public Health, Experimental and Forensic Medicine, University of Pavia, Pavia, Italy

**Keywords:** neuromuscular disorder, creatine kinase, exercise tailoring, resistance training, aerobic training

## Abstract

**Background:**

Patients with central core myopathy (CCM) can be at risk of exercise-induced rhabdomyolysis and myalgia. Despite its possible positive effects, physical training has been long avoided in these patients as no population-specific exercise adaption strategies have been developed. Here we present the case of a 17-year-old male CCM patient who underwent a 3-month training program tailored to a preliminary test aimed at assessing his physical exertion tolerance measured via changes in serum creatine kinase (CK).

**Methods:**

The preliminary tolerance test consisted of three 25-minute sessions (one session per week) of physical exercise (aerobic, resistance and mixed) at an intensity quantified as level 6 of the Borg Category Ratio (CR) 0–10 scale. A blood sample to assess CK was conducted 36 h following eachsession. The intervention consisted of a training program (three sessions per week) including both resistance and aerobic exercises concomitant with a personalized nutritional plan. Before and after intervention, a battery of metabolic (indirect calorimetry, bioimpedance) and cardiopulmonary (CPET) tests were performed.

**Results:**

After training, improvements of the anaerobic threshold (+6.9%), normalized VO_2_ max (+15%) and body composition (muscle mass, +1.1 kg; fat mass, −1.1 kg were observed without pain, rhabdomyolysis, and blood CK augmentation compared to pretraining values.

**Conclusion:**

Our results highlight that a mixed aerobic/resistance training, properly tailored and supported by a specific nutritional plan, may safely improve the physical fitness and body composition in a CCM patient. Dosing exercise-induced CK serum change following Borg CR-10 intensity assessment, may be useful to correctly tailor physical exercise in these patients.

## 1 Introduction

Central core myopathy (CCM) is a rare (prevalence of 1–9 cases per 1,000,000) inherited congenital neuromuscular disorder caused by pathogenic variants to the gene (*RYR1*) that encodes the ryanodine receptor (RyR1) (Robinson et al., 2006; Jungbluth, 2007; Orphanet, 2023). Peculiar feature of the pathology is the presence of areas along the muscle fibre’s longitudinal axis (central cores) characterized by the absence of oxidative enzymatic activity due to mitochondrial depletion owing to intracellular calcium dysregulation (Jungbluth, 2007). The clinical manifestations of the disease are heterogeneous; it typically begins during the postnatal period with hypotonia and widespread muscle weakness which is generally distributed proximally with major involvement of the pelvic girdle and axial muscles (Topaloglu, 2020). Adjunctive manifestations can be a high serum creatine kinase (CK) value (hyperCKemia), exertional myalgia, rhabdomyolysis, periodic muscle rigidity and paralysis, while some subjects remain asymptomatic (Topaloglu, 2020). Since a disease-modifying treatment is not available yet, it is of major importance to identify strategies aimed at containing or, possibly, counteracting the structural and functional muscle deficits dictated by the disease.

Exercise is suggested to reduce physical deteriorationin several neuromuscular pathologies including muscular dystrophies (Voet et al., 2019), metabolic myopathies (Parimbelli et al., 2021), and critical illness polyneuropathy and myopathy (Crisafulli et al., 2022). To date only two case reports reporting the effect of aerobic activity (Hagberg et al., 1980; Schreuder et al., 2010) in patients with CCM are available, while resistance training, on a theoretical basis, is often avoided (Jungbluth, 2007). As CCM is a myopathy with a severe mitochondria impairment, the lack of available literature exploring the effects of physical activity in this population is most likely attributed to the potential risks for metabolic stress and exercise related muscle damage followed by rhabdomyolysis and myoglobinuria. However, the identification of the risks and related preventive measures could make physical exercise also usable by this population. For instance, the high risk of exercise-induced muscle damage dictated by the pathology, could be addressed by an adapted physical training structured in compliance with the individual tolerance to physical effort. Moreover, as in healthy subjects, the adequate control of physiological factors known to interplay with the muscular plastic response may be critical to guarantee a certain degree of adaptation to exercise. In particular, inadequate nutrition (over/undernutrition, and/or poor-quality nutrition), which has a major impact on morbidity and mortality in neuromuscular disease (Motlagh et al., 2005), may represent a confounding factor impacting on the overall effects of training in this condition. The adjustment of the calorie intake to the daily energy expenditure and providing the correct macronutrients proportion and macro/micronutrients quality in the diet could be a valuable strategy to optimize the training outcomes.

Here, we present a CK tailored physical training program associated to a personalized nutritional plan in a 17-year-old boy affected by CCM. Before and after the training program, the patient underwent a battery of blood, metabolic and cardiopulmonary tests to examine training adaptations.

## 2 Case report

**FIGURE 1:**
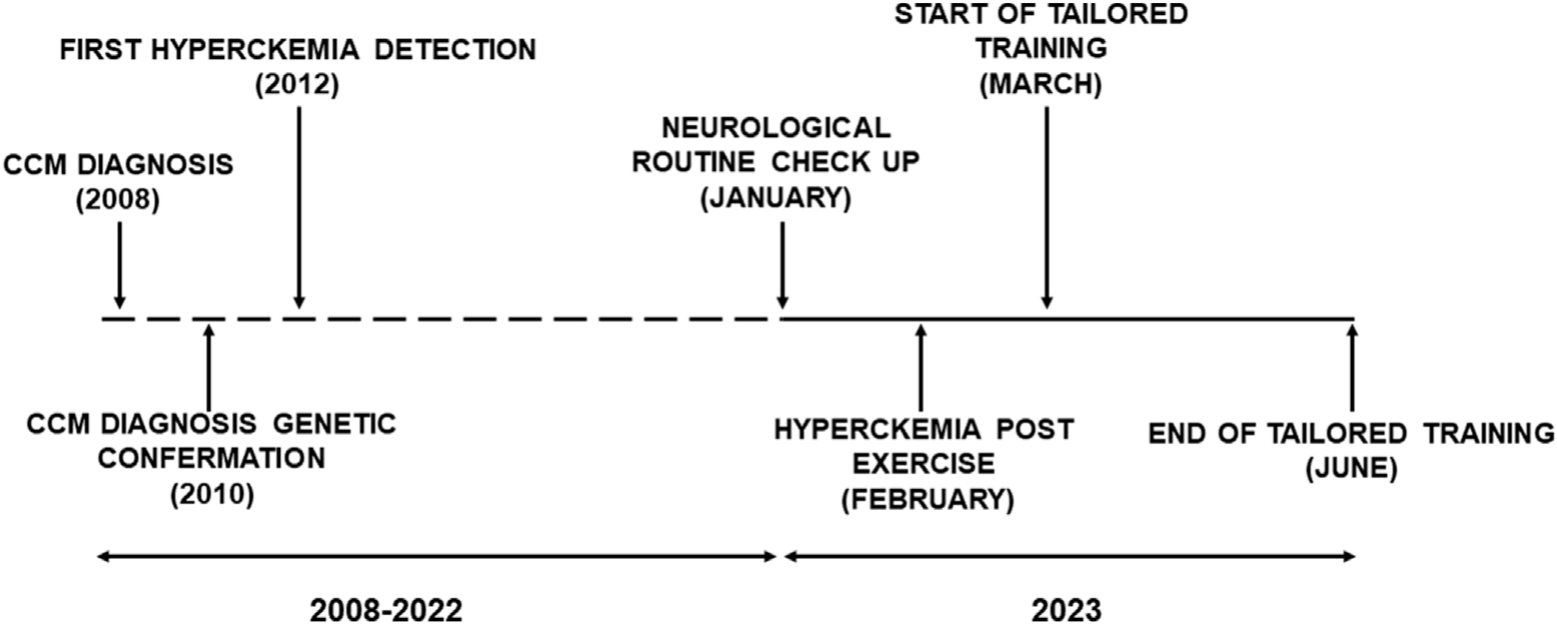
Timeline of case’s salient events.

The patient was a 17-year-old male affected by CCM (presenting the c.7354C>T variant of gene *RYR1* exon 46), without associated pathologies. His weight was 53.3 kg, and his height was 1.56 m. He was diagnosed with CCM when he was 3 years old, and a subsequent genetic examination (Figure 1) highlighted a heterozygous pathogenic variant in a gene compatible with the patient’s clinical manifestations, which leans towards the diagnostic classification of CCM pathogenic variant of the dominant gene *RYR1*. In January 2023, he underwent a routine neurological check reporting an increased perception of fatigue, which negatively impacted his ability to perform school-based physical activities. The clinical examination highlighted the patient’s maintained ability to change position from laying supine to sitting, an independent physiological gait as well as the ability to jump in single and double stance, albeit with little elevation. The spine appeared aligned and normally flexible. Muscle trophism, tone and reflexes were within normal limits in the patient’s upper limbs, shoulder girdle, pelvic girdle, and lower limbs. Muscle strength was within normal limits in the upper limbs and shoulder girdle, whereas a loss of strength was observed in the adductor and gluteal muscles during assessment of the pelvic girdle and lower limbs. Further, the triceps surae were observed to be pseudo-hypertrophic. Magnetic resonance imaging (MRI) showed bilateral adipose infiltration in the hamstrings, sartorius and triceps surae muscles and a marked bilateral atrophy of the gluteal muscles. The semitendinosus and rectus femoris muscles were partially spared.

At the end of this clinical routine, the patient was advised at our sport medicine centre.

### 2.1 Clinical framework and hyperCKemia investigation

The participant and his parents signed a written informed consent form before his inclusion in the study. The patient’s clinical history highlighted fundamental critical episodes. In 2012 a serum hyperCKemia (around 1,500 U/L) was measured in the emergency room following a car accident. Considering that hyperCKemia can be associated with CCM (Topaloglu, 2020), further CK measurements were recommended to tell if this incidental evidence had to be attributed to the underlying disease or to the car accident, confirming that these abnormal values were the norm for the patient. Therefore, since this event, periodical CK investigations were performed to keep the parameter under observation. In February 2023 a marked increase in serum CK (7604 U/L) was found 2 days after at home unsupervised physical activity as follows: 1) the activity was performed every day for 6 days; 2) the total duration of each exercise session was 25–30 min; 3) the activity was alternated: 1 day biking, 1 day body weight resistance training with squat and plank paradigms (approximately 30 s per exercise); 4) on the second day, delayed onset muscle soreness (DOMS) appeared and persisted in the following 7 days; 5) to retrospectively quantify the perceived effort at the end of each session, the Borg category ratio (CR) 0–10 scale (Borg, 1982) was used, given its proven effectiveness in quantifying effort in both resistance training (Hiscock et al., 2015; Morishita et al., 2019) and aerobic training (Marinov and Kostianev, 2003; Yáñez-Sepúlveda et al., 2018). The value reported by the subject was eight for each session. These data were subsequently considered to tailor the training program.

### 2.2 CK values as a criterion for training program tailoring

In March 2023, we aimed to find a safe and effective strategy to incorporate combined aerobic and resistance exercise into the patient’s life in an attempt to counteract the progression of disease. Suchchoice was based upon the documented role of aerobic and resistance exercise in preserving muscle structure and function in both healthy (Egan and Zierath, 2013) and diseased muscle (Voet et al., 2019; Parimbelli et al., 2021; Crisafulli et al., 2022). The following descripted methodologies are part of the clinical procedures habitually adopted in our sport medicine centre. Notably, we usually apply such paradigms in patients with other myopathies at risk of rhabdomyolysis, such as metabolic myopathies. To safely provide a tailored training program, adapted from the independent physical activity reported by the patient, the subject underwent a 3-week (one exercise session per week) preliminary tolerance test in which exercise intensity was reduced (Borg 8→Borg 6) and duration was maintained (25 min) as follows: week 1, aerobic training on a bike alternating 2 min at Borg 4 (heart rate range, 117–127 bpm) and 1 min at Borg 6 (heart rate range, 135–150 bpm), the training was carried out with the use of a heart rate monitor; week 2, resistance training including exercises for core and abdominal muscle [hand-foot plank, front plank (2 sets for 30 s each) crunch and reverse bent leg crunch (2 sets of 15 rep each)], lower limbs [squat, two-legged and single-legged calf standing, climb a step with weight (3 sets of 12 reps each)] and upper limbs including the shoulders (overhead press, lateral raises), and triceps and pectorals muscle (push-ups) (2 sets of 12 reps each). In this session, exercises were interspersed with 2 min recovery and performed without emphasizing the eccentric phase of the contraction, known as potential cause of rhabdomyolysis (Heled et al., 2005); week 3, mixed aerobic [10 min of bike alternating 2 min at Borg 4 (heart rate range, 117–127 bpm) and 1 min at Borg 6], and resistance training (repeating week 2 session removing 1 set and 2 repetitions from each exercise). All sessions were supervised by a qualified operator (MV) for patient safety and to ensure compliance with the proposed program.

Thirty-six hours following each exercise session, a blood sample was collected to measure circulating CK levels. For 1 week after each session, a daily quantification of soreness was obtained by means of the visual analogue scale (VAS), commonly used to quantify DOMS (Dannecker et al., 2003; Gast et al., 2023). DOMS peak value and duration were reported.

At the completion of the 3-week preliminary tolerance test, the resulting CK and DOMS measures were as follows: week 1, CK = 1343 U/L, DOMS duration (days) = 0, DOMS peak = 0; week 2, CK = 1145 U/L, DOMS duration (days) = 1, DOMS peak = 1 (1 day after test); week 3 (mixed aerobic and resistance training), CK = 1377 U/L, DOMS duration (days) = 1, DOMS peak = 1 (1 day after test).

### 2.3 Training program

**FIGURE 2:**
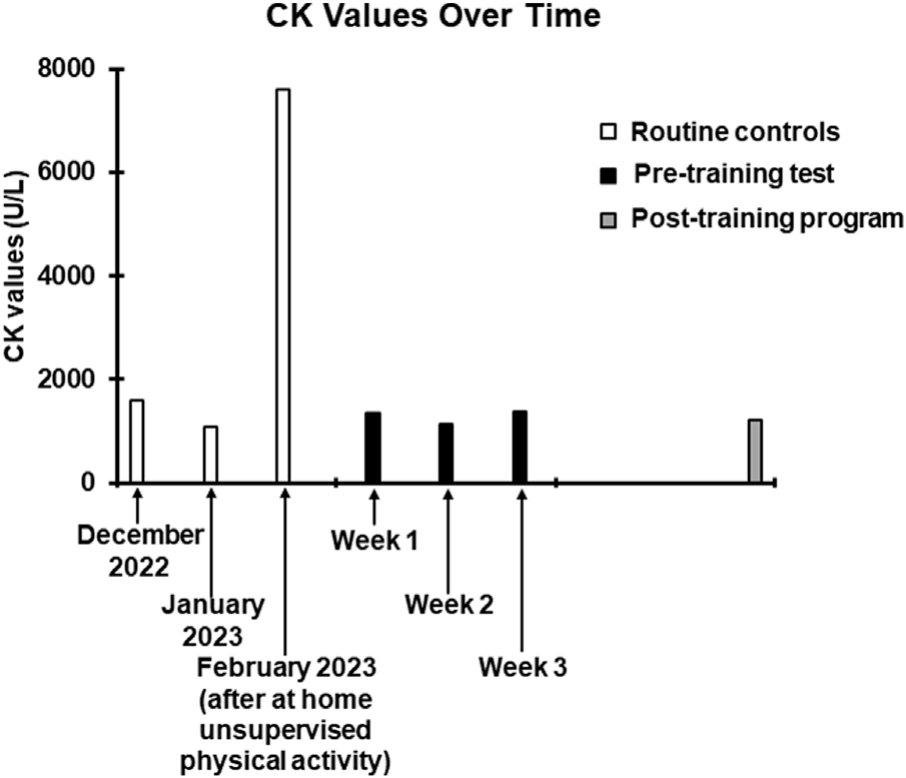
Trend of CK values over time. The white columns represent the values obtained in the last three routine checks carried out before contacting our centre. The December 2022 and January 2023 values have been entered to show data relating to the patient’s normal CK values. The February 2023 value, as specified, was obtained following the at home unsupervised physical activity. The black columns refer to the values obtained 36 h after the training sessions of the preliminary test. The grey column refers to the value obtained 36 h after the last session of the training program.

The training program included resistance exercises twice a week and aerobic exercises once a week, which were carried out with the same modalities of the preliminary test but with less rest days between the sessions (from 7 days to 2 days). The weekly frequency of the sessions (3 per week) was chosen considering that 36 h after the preliminary training sessions, time frame within which, considering the moderate intensity of the exercises, serum CK should have reached its peak (Clarkson et al., 1992), the CK values were comparable to those found in some previous routine checks (Figure 2), suggesting a possible cumulative effect as unlikely. Moreover, the reported modest duration and degree of DOMS, indicated the reasonable possibility of good tolerability of the proposed workload, without inducing exercise-related pain or discomfort. The training program lasted 3 months as it is a time frame widely sufficient to induce adaptations to both resistance (Damas et al., 2018) and aerobic training (Matsuo et al., 2014; Duarte et al., 2023). Stretching followed each training session to prevent contractures and strains (Harvey and Herbert, 2002; Nunes et al., 2020) which, given the predisposition dictated by the disease (Lawal et al., 2020), could likely have occurred. As during the preliminary test, all sessions were carried out under supervision.

### 2.4 Metabolic and functional evaluation

Before and after the training program, the patient underwent a battery of metabolic and cardiopulmonary tests. Resting metabolic rate (RMR) was measured at 08.00 h in a room where ambient temperature was fix at 23°C using indirect calorimetry technique (Quark PFT, Cosmed, Italy). The patient arrived in a fasted state (from 8 h) and was instructed to lie quietly for 10 min, after which a ventilated hood was placed over the head (Canopy, Cosmed, Italy). The subject was asked to relax but remain awake for the duration of the test (25 min). The dilution rate was adjusted during the first 5 min of the test so the fraction of expired VCO_2_ was between 1.0% and 1.2%. This portion of the test was excluded to allow breathing and dilution rate to normalize; RMR and RQ were averaged over the remaining 20 min. Initial RMR was 1,588 kcal/day, with an RQ of 0.76 (normal range at rest 0.7–0.85) (Guthrie et al., 1964), highlighting a high percentage of fat utilization (83%).

Bioimpedance (Akern, Pontassieve, Italy) was utilized to evaluate body composition through the measurement of its impedance consisting of resistance (R) and reactance (Xc). The following parameters were measured: muscle mass (MM), body cell mass (BCM), fat mass (FM) and phase angle (PA, 6) measured as [arctangent (Xc/R) × 180°/π]. For the measurement of MM, BCM and FM values, bioimpedance uses predictive equations that have not been validated in this type of patient and could, therefore, be altered by over- or underestimation errors (Kyle et al., 2004; Beaudart et al., 2020). For this reason, considering that the bias, if present, would be constant, the pre and post training evaluation relating to these parameters is proposed only as the difference between the values obtained before and after the intervention (see Table 1).

**TABLE 1:** Absolute and relative of the functional parameters measured at the beginning and at the end of the training program intervention.

Functional tests and parameters	Pre-training program	Post-training program	Absolute change	Relative change (%)
Indirect calorimetry				
RMR (Kcal/day)	1,588	1,613	+25	+1.6
Respiratory quotient	0.76	0.74	−0.02	−2.6
Bioimpedance				
Phase Angle (°)	6	6.5	+0.5	+8.3
Body weight (Kg)	53.3	52	−1.3	−2.4
Fat mass (Kg)	—	—	−1.1	—
Muscle mass (Kg)	—	—	+1.1	—
Body cell mass (Kg)	—	—	+1	—
CPET				
VO_2_ max (mL/min)	1,703	1,910	+207	+12.2
VO_2_ max/BW (mL/min/Kg)	31.9	36.7	+4.8	+15
Ventilatory Anaerobic threshold (mL/min)	1,241	1,327	+86	+6.9
Respiratory exchange ratio (VCO_2_/VO_2_)	1.15	1.15	0	0
Maximal Borg value	10	10	0	0
Blood Lactate (mmol/L)	10	9.3	−0.07	−7
VE/VCO_2_	29.1	26.8	−2.3	−7.9

RMR, resting metabolic rate; Kcal, kilocalories; Kg, kilograms; CPET, cardiopulmonary exercise test; ml, millilitres; min, minute; BW, body weight; VCO_2_, volume of carbon dioxide produced; VO_2_, oxygen consumption; mmol, millimoles; L, liters; VE, ventilation.

A cardiopulmonary exercise test (CPET) was performed on a cycloergometer (E 100, Cosmed, Italy), with the patient wearing a two-way breathing mask covering the nose and mouth (V2 Mask TM, Hans Rudolph Inc., United States), connected to a gas analyzer (Quark PFT, Cosmed, Italy). CPET was performed with an incremental technique (15 W every 2 min, with a previous warm up of 5 min at 20 W), and VO_2_ and VCO_2_ output were measured using the breath-by-breath method. The test was performed until exhaustion, with the achievement of a respiratory exchange ratio (RER) of 1.15 (>1.1 maximal effort index), a blood lactate value of 10 mmol/L and a Borg CR 10 value of 10. For blood lactate determination, blood sample was obtained from the earlobe and concentration was determined by a specific lactate detection device (Lactate Pro 2, Arkray, Kyoto, Japan). Ventilatory anaerobic threshold (VAT) was assessed from the slopes of VO_2_ and VCO_2_.

VO_2_ max was 1,703 mL/min corresponding to 31.9 mL/min/kg (normal range for age and gender over 1,933 mL/min or over 24.8 mL/min/kg). Overall CPET underlined a very low oxygen uptake during the effort, not from respiratory deficiency, as VE/VCO_2_ was below 30, but from the mitochondrial metabolic impairment. All the pre- and post-training program values are reported in Table 1. Coherently with the preliminary tolerance test procedure, post-training program CK measurement was performed 36 hours after the last exercise session (Figure 2).

### 2.5 Nutritional plan

To plan an adequate nutritional support to the training program, a qualified team led by an experienced dietitian (EL) assessed the nutritional status of the patient by using diagnostic-clinical tools including the evaluation of the dietary intake (macronutrients, micronutrients and energy intake), blood biochemical analysis, and anthropometrical and body composition determinations (Taberna et al., 2019). The patient’s long-term diet was assessed by using the Quantitative Food-frequency Questionnaire (FFQ) on a typical week from which three non-consecutive days were extrapolated (Yang et al., 2010). The nutritional intake adequacy was then analysed based on the Recommended Levels of Nutrients and Energy Intakes (LARN) (Sinu, 2023). Energy intake and macronutrient distribution resulted inadequate to satisfy the needs (3,130 kcal/day; CHO 43%, FAT 35%, daily proteins 107.13 gr). An elevated consumption of saturated fats was also found. Blood biochemical evaluation revealed vitamin C (4.3 mg/L), vitamin D (23.6 ug/L) and Cu (60 ug/dL) deficiencies.

Considering a RMR of 1,588 kcal/day (Table 1), a personalized isocaloric Mediterranean diet of 2,000 kcal/day was determined based on the estimated calorie needs per day, by age and gender in patients with neuromuscular disorders (Burgos et al., 2018). The daily diet was tailored to reach a macronutrient intake of 50% CHO, 30% FAT, and a protein intake of 1.5 g/kg body weight/day in accordance with nutritional recommendations for the management of sarcopenia (Rogeri et al., 2021). In addition, the patient began a regimen of nutritional supplementation to sustain protein synthesis (Aminotrofic, Errekappa, 7 g/day of essential amino acid mixture) and to adjust the identified vitamin deficiencies (multivitamin Unicomplex Plus, Kline, 2 sachets/die). This nutritional regimen was maintained all throughout the training program and the compliance was assessed via a diary in which the patient had to record the food consumed. Before every third training session of the week, the diary was checked by one of the researchers responsible for the nutritional plan (JL, EL, MN, SA).

## 3 Discussion

In author’s knowledge, this is the first time in which: 1) feasibility, safety, and effectiveness of a training program, based on combined endurance and resistance exercises and supported by an *ad hoc* nutritional plan is tested in a CCM patient; 2) a CK-Borg 10 CR approach is used to tailor the correct exercise program in a myopathic patient.

Our results revealed that 3 months of adapted physical training, were sufficient to determine a significant improvement of the patient’s aerobic fitness and body composition.

At the end of training, an increase in normalized VO_2_ max (from 31.9 to 36.7 mL/min/kg) and an associated enhancement of the VAT(from 1,241 to 1,327 mL/min) were observed. VO_2_ max is notoriously determined by combined cardiac output and mitochondrial oxidative capacity of the skeletal muscles (Betik and Hepple, 2008). Hence, the observed VO_2_ max improvement acquires relevance if contextualized with respect to the clinical picture and the physiopathology of the disease, which is characterized by an abrupt reduction of mitochondria in the skeletal muscle (Talwalkar et al., 2006).

An improvement in VO_2_ max following physical training has been reported in several populations of subjects (Mandroukas et al., 1984; Hedermann et al., 2016; Curcic et al., 2017), including patients carrying mitochondrial impairments (Jeppesen et al., 2006). However, in author’s knowledge, only one previous case report has been conducted to test the effectiveness of the exercise alone on the aerobic fitness in a patient with CCM (Hagberg et al., 1980). In fact, in a second available case report (Schreuder et al., 2010), a combined aerobic-pharmacologic (beta-adrenergic agonistic, Albuterol) treatment was tested in a child with CCM, denying the possibility to discriminate the quote of exercise and pharmacological contribution to the achieved improvement in aerobic capacity. In the study by Hagberg et al. (1980) a 36-year-old male CCM patient reported a significant increase in VO_2_ max after 9 months of endurance training, associated with an improvement in some activities of daily living. Although subjects’ age (17 vs. 36 years old), exercise modality (resistance and aerobic vs. only aerobic) and training time (3 vs. 9 months) are quite different, our result would confirm the same findings.

Despite the potential risk of hyperCKemia and rhabdomyolysis associated with resistance training in a patient with CCM, we have been able to show that the incorporation of this mode of exercise training can be done safely, providing the rationale for testing such approach in a larger sample of patients. Tailoring the exercise intensity based on a combined CK-Borg CR value approach proved to be successful to set both the resistance and aerobic training exercises on safe and effective volumes. In fact, the CK level found after 3 months of training was unchanged compared to pretraining values suggesting that proposed training protocol was not associated to cumulative fibre damage and further CK leaking (see Figure 2).

Indeed, a significant amelioration in body composition was observed as demonstrated by a body weight reduction, a decrease in fat mass, and an increased muscle mass and body cell mass (Table 1). Although no evidence is currently available regarding the effect of resistance training in CCM, the observed increase in muscle mass and the slight increase in RMR (Speakman and Selman, 2003; Johnstone et al., 2005) likely indicate that the combination of resistance training and nutritional rebalancing can be a valid tool in counteracting muscle loss in these patients. This observation is in line with what has been reported for other subjects’ populations (Montero-Fernández and Serra-Rexach, 2013; He and Ye, 2020). On the other hand, the negative change in fat mass may mirror the effects of the adopted integrated nutritional and training program (Lee and Lee, 2021) as aerobic (Willis et al., 1985) and resistance training (Wewege et al., 2022) as well as in presence of an isocaloric diet (Kim et al., 2014) all contribute to this outcome. Finally, the observed increase in phase angle following training (6°→6.5°) may mostly reflect an improved cell membranes integrity and functionality, intracellular composition, and a greater tissue capacity (Mattiello et al., 2020).

## 4 Concluding remarks and key points

The effectiveness of physical exercise as a possible therapeutic tool in maintaining skeletal muscle structure and function in several neuromuscular disease (NMD) seems to be confirmed in a single case of CCM. Overall, the presented case underlines the importance of personalizing physical exercise intensity and frequency and nutrition with respect to the clinical condition of the patient. In fact, independent, unsupervised, and uncontrolled physical activity may lead to a potentially dangerous hyperCKemia as observed in the clinical history of this subject.

Our results, which refer to a single case, are obviously not generalizable to other patients suffering from CCM. Moreover, another limitation regards the specific contribution of training and diet to the observed positive changes in body composition. However, our data show that a training program including both aerobic and resistance exercises, supported by a specific nutritional plan, may be a safe and effective instrument to ameliorate the structural and functional condition in this kind of patient. This also suggests that exercise guidance may become a pivotal component of future clinical care guidelines for *RYR1*-related myopathies.

Reasonably, careful tailoring of exercise intensity and periodization based on objective (CK) and subjective (Borg CR 10) measurements could be an effective strategy also valid in other NMD at risk of rhabdomyolysis, making physical exercise feasible for many patients for whom, to date, it is precluded.

## Data Availability

The original contributions presented in the study are included in the article/Supplementary Material, further inquiries can be directed to the corresponding author.
